# Photoluminescent Gold Nanoclusters in Cancer Cells: Cellular Uptake, Toxicity, and Generation of Reactive Oxygen Species

**DOI:** 10.3390/ijms18020378

**Published:** 2017-02-10

**Authors:** Marija Matulionyte, Dominyka Dapkute, Laima Budenaite, Greta Jarockyte, Ricardas Rotomskis

**Affiliations:** 1 Biomedical Physics Laboratory, National Cancer Institute, P. Baublio st. 3b, Vilnius LT-08406, Lithuania; dominyka.dapkute@nvi.lt (D.D.); budenaite.laima@gmail.com (L.B.); greta.jarockyte@nvi.lt (G.J.); 2 Biophotonics Group of Laser Research Centre, Vilnius University, Sauletekio ave. 9, Vilnius LT-10222, Lithuania

**Keywords:** photoluminescence, gold nanoclusters, breast cancer cells, accumulation, toxicity, reactive oxygen species

## Abstract

In recent years, photoluminescent gold nanoclusters have attracted considerable interest in both fundamental biomedical research and practical applications. Due to their ultrasmall size, unique molecule-like optical properties, and facile synthesis gold nanoclusters have been considered very promising photoluminescent agents for biosensing, bioimaging, and targeted therapy. Yet, interaction of such ultra-small nanoclusters with cells and other biological objects remains poorly understood. Therefore, the assessment of the biocompatibility and potential toxicity of gold nanoclusters is of major importance before their clinical application. In this study, the cellular uptake, cytotoxicity, and intracellular generation of reactive oxygen species (ROS) of bovine serum albumin-encapsulated (BSA-Au NCs) and 2-(*N*-morpholino) ethanesulfonic acid (MES)-capped photoluminescent gold nanoclusters (Au-MES NCs) were investigated. The results showed that BSA-Au NCs accumulate in cells in a similar manner as BSA alone, indicating an endocytotic uptake mechanism while ultrasmall Au-MES NCs were distributed homogeneously throughout the whole cell volume including cell nucleus. The cytotoxicity of BSA-Au NCs was negligible, demonstrating good biocompatibility of such BSA-protected Au NCs. In contrast, possibly due to ultrasmall size and thin coating layer, Au-MES NCs exhibited exposure time-dependent high cytotoxicity and higher reactivity which led to highly increased generation of reactive oxygen species. The results demonstrate the importance of the coating layer to biocompatibility and toxicity of ultrasmall photoluminescent gold nanoclusters.

## 1. Introduction

Bioimaging is one of the frontiers in biomedical sciences and has significant impact in clinical and medical research. In the past decade, noble metal nanoclusters (NCs), especially those synthesized from gold, with diameters below 2 nm have emerged as fascinating fluorescent nanomaterials and attracted considerable interest in both fundamental biomedical research and practical applications as very promising photoluminescent agents for biosensing [1,2], bioimaging [3,4], and targeted therapy [5,6,7,8]. Due to enhanced quantum confinement of free electrons as the sizes of nanoclusters become comparable to the electron Fermi wavelength (~0.5 nm for gold) [9,10,11] ultrasmall gold NCs no longer possess localized surface plasmon resonance [12] and instead exhibit molecule-like behaviors [13], such as tunable photoluminescence, the emission wavelength being dependent on the number of atoms in the cluster [13]. To date, several different synthetic strategies have been developed for the formation of photoluminescent gold NCs. Synthesis using thiol-containing small-molecules as capping agents are the most common as strong Au-S interactions on the nanocluster surface lead to highly stable Au NCs [14,15]. However, growing demand for facile and eco-friendly synthesis brought up other kinds of weak reducing agents, such as Good’s buffers [16]. Increasing interest in “green” synthesis further led to development of photoluminescent gold nanoclusters synthesized using biomolecules such as proteins, peptides, and DNA to combine unique optical properties of Au NCs and biological functions of the biomolecules [17]. One example is a new class of photoluminescent gold nanoclusters synthesized within the protein templates, the first synthesis report being on the bovine serum albumin (BSA)-stabilized photoluminescent gold nanoclusters proposed by Xie et al. [18]. Other proteins such as insulin [19,20], human transferrin [21], ferritin [22], hemoglobin [23], trypsin [24], and lysozyme [25] have also been shown to successfully serve as templates for the formation of photoluminescent Au NCs.

Despite the great improvement in the field of Au NCs synthesis, interaction of cells with ultra-small nanoclusters remains poorly understood. The cellular uptake and toxicity of large Au nanoparticles vary greatly depending on the size [26,27], shape [28], coating ligands [29] and surface charge [26,30]. Several authors have shown that gold nanoparticles smaller than 50 nm efficiently accumulate in cells [26,31,32], however, the results are limited as the sizes of NCs decrease below 2 nm. Moreover, there are numerous contradictory results on cytotoxicity/proliferation-promoting effects of gold nanoparticles (Au NPs). Pan et al. reported highly increased cytotoxicity of ultrasmall (1.4 nm) gold nanoclusters in comparison with cytotoxicity of other 0.8–15 nm size gold nanoparticles [33]. Yet when small Au NPs aggregate and form solid plates, they were found to be not toxic to the cells but instead promoted growth [34]. Similar results were observed as cells were incubated with 70 nm size silica-gold core-shell structure [35] or seeded on a gold film with 24 nm gold nanoparticles immobilized on top [36]. However, as one of a few studies on cytotoxicity of ultrasmall photoluminescent Au NCs has shown, capping agents play a very important role in biological activity of Au NCs [37]. Therefore, it is of major importance to assess the biocompatibility and potential toxicity of Au NCs before their clinical application as well as to evaluate possible mechanisms of toxicity. 

One of the primary reasons for toxicity caused by nanomaterials has found to be reactive oxygen species (ROS) [38,39,40,41]. Chemically reactive molecules such as hydroxyl radicals (^•^OH), superoxide anions (O_2_^•−^), and hydrogen peroxide (H_2_O_2_), are continually being generated via the process of aerobic cellular metabolism. At low levels, ROS may play an important role in molecular signaling, regulating fundamental biological processes such as cell viability, proliferation, migration, and differentiation [42,43]. However, overproduction of ROS may potentially result in stress response and interference with basic cellular functions, which further leads to DNA damage, unregulated cell signaling, change in cell motility, cytotoxicity, and apoptosis [41,44]. 

Herein, we selected biocompatible BSA-encapsulated photoluminescent Au NCs to compare Au NCs synthesized within protein template and ultrasmall 2-(*N*-morpholino) ethanesulfonic acid (MES)-capped photoluminescent Au NCs as a model for “naked” Au NCs due to their very thin coating layer. The cellular uptake and cytotoxicity of Au NCs along with intracellular generation of reactive oxygen species in MCF-7 and MDA-MB-231 breast cancer cells was investigated. 

## 2. Results

### 2.1. Spectrometric Characteristics of Au NCs

**Figure 1:**
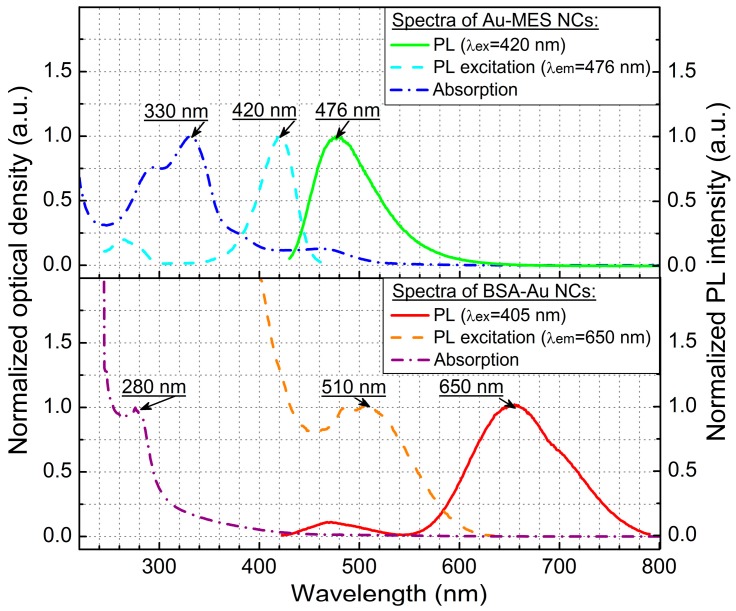
Normalized absorption, photoluminescence (PL) and photoluminescence excitation spectra of 2-(*N*-morpholino) ethanesulfonic acid (MES)-capped photoluminescent gold nanoclusters (Au-MES NCs) (top) and bovine serum albumin-encapsulated gold nanoclusters (BSA-Au NCs) (bottom) in deionized water.

Absorption, photoluminescence, and photoluminescence excitation spectra of freshly synthesized BSA-Au NCs and Au-MES NCs are presented in Figure 1. 

Absorption spectrum of synthesized Au-MES NPs (Figure 1, dash-dot blue line) had a main peak with a maximum at 330 nm, two peaks of lower absorbance at 290 and 475 nm and one less expressed band at around 390 nm. Components used for the synthesis did not have absorption bands in these spectral regions [45]. Photoluminescence of Au-MES NCs with a maximum at 476 nm wavelength (λ_ex_ = 420 nm) (Figure 1, green solid line) was detected. Photoluminescence excitation spectrum registered at photoluminescence maximum (λ_em_ = 476 nm) (Figure 1, cyan dashed line) had a main maximum at 420 nm and another band of lower intensity at around 260 nm wavelength. In the previous report, we have shown that according to the calculations, based on free electron gas (Jellium) model [46], Au NCs that exhibit photoluminescence at 476 nm wavelength consist of ~9 gold atoms. The size of the NCs should be less than 1 nm, and ~0.5 nm size NCs were measured using atomic force microscope [45].

The absorption spectrum of BSA-Au NCs (Figure 1, dash-dot purple line) exhibited gradually increasing absorbance to the shorter wavelength spectral region with a distinct absorption band at 280 nm. The shape of the spectrum coincides with the absorption spectrum of pure BSA [47]. Photoluminescence spectrum of BSA-Au NCs solution (λ_ex_ = 405 nm) has a main peak with a maximum at 650 nm and another band of lower intensity at 468 nm (Figure 1, red solid line). Components used for the synthesis of BSA-Au NCs (HAuCl_4_, BSA) or the mixture of those two materials did not exhibit photoluminescence in red spectral region (600–700 nm) [47]. BSA-Au NC solution has a wide photoluminescence (PL) excitation spectrum (λ_em_ = 650 nm) (Figure 1, orange dashed line) gradually increasing to the shorter wavelength spectral region with a distinctive band at around 510 nm wavelength. The hydrodynamic size of BSA-Au NCs is 9.4 nm on average, the core Au NCs being composed of ~29 gold atoms as it was calculated using the free electron gas model in our previous report [47].

### 2.2. Accumulation of Au NCs in Live Cancer Cells

**Figure 2:**
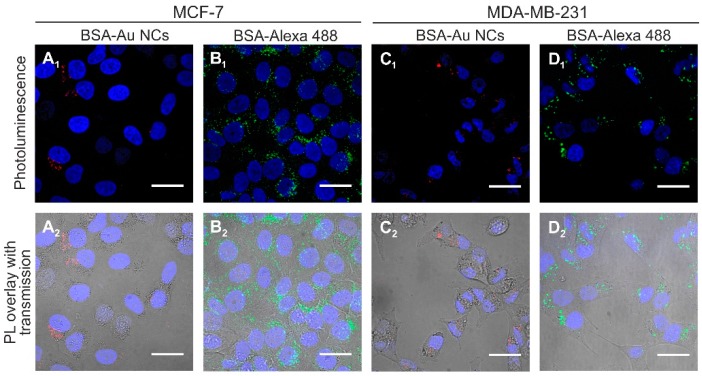
Accumulation of photoluminescent BSA-Au NCs (λ_ex_ = 488 nm) (**A_1_**,**A_2_**,**C_1_**,**C_2_**) and BSA-Alexa conjugate (λ_ex_ = 488 nm) (**B_1_**,**B_2_**,**D_1_**,**D_2_**) in MCF-7 and MDA-MB-231 cells after 24 h of incubation, nuclei stained with Hoechst 33258 (λ_ex_ = 405 nm). Scale bar is 30 μm.

**Figure 3:**
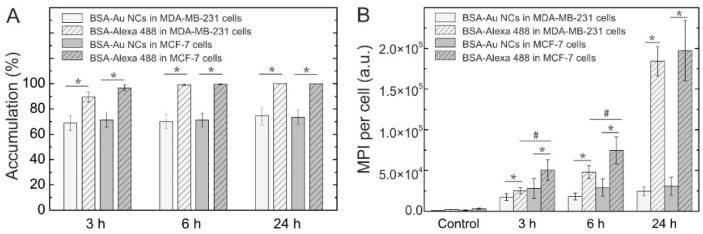
Accumulation of photoluminescent BSA-Au NCs and BSA-Alexa conjugate in MCF-7 and MDA-MB-231 cells after 3, 6, and 24 h of incubation. Percentage of the cells that have accumulated BSA-Au NCs and BSA-Alexa conjugate (**A**); mean PL intensity (MPI) of BSA-Au NCs and BSA-Alexa conjugate per cell (**B**). Control represents autofluorescence of non-treated cells. Error bars show the standard deviations. * indicates significant differences between accumulation of BSA-Alexa 488 and BSA-Au NCs (*p* ≤ 0.05); ^#^ indicates significant differences between the MCF-7 and MDA-MB-231 cell lines (*p* ≤ 0.05).

To investigate internalization of BSA-Au NCs and BSA in MCF-7 and MDA-MB-231 breast cancer cells, the cells were incubated with BSA-Au NCs (56 mg/mL) and with BSA-Alexa 488 conjugate (0.01 mg/mL) respectively. After 24 h of incubation BSA-Au NCs were observed accumulated in vesicles inside MCF-7 cancer cells (Figure 2A_1_,A_2_). No fluorescence at this spectral region was observed in the nuclei of the cells. BSA-Au NCs did not accumulate uniformly, flow cytometry data showed that only 73.5% of the MCF-7 cells had internalized BSA-Au NCs after 24 h of incubation (71.3% after 3 h and 6 h of incubation respectively) (Figure 3A). For comparison, after 24 h of incubation the vesicles containing fluorescent BSA-Alexa 488 conjugate were observed in all MCF-7 cells (Figure 2B_1_,B_2_). 

Flow cytometry data confirmed that 100% of the cells had internalized fluorescent BSA-Alexa 488 conjugate after 24 h of incubation (96.6% and 99.5% after 3 h and 6 h of incubation, respectively) (Figure 3A). Accumulation of photoluminescent BSA-Au NCs (λ_ex_ = 488 nm) and BSA-Alexa conjugate (λ_ex_ = 488 nm) in MDA-MB-231 cells was very similar (Figure 2C_1_,C_2_,D_1_,D_2_). After 3, 6, and 24 h of incubation 68.8, 70.0, and 74.6% of cells had internalized BSA-Au NCs. For comparison, 89.4, 99, and 100% of MDA-MB-231 cancer cells had internalized BSA-Alexa 488 conjugate after 3, 6, and 24 h of incubation, respectively (Figure 3A). Mean photoluminescence intensity (MPI) values of BSA-Au NCs and BSA-Alexa conjugate per cell were also analyzed. The results have shown that MPI of the internalized BSA-Au NCs per cell does not increase over time in comparison with MPI after 3 h of incubation in both MCF-7 and MDA-MB-231 cells (Figure 3B). On the contrary, MPI of the BSA-Alexa conjugate per cell after 6 and 24 h of incubation increased respectively 1.5 and 3.9 times in comparison with MPI after 3 h of incubation in MCF-7 cells. The difference was even higher for MDA-MB-231 cancer cells—the MPI of the BSA-Alexa conjugate per cell increased over time 1.9 and 7.3 times after 6 and 24 h of incubation, respectively.

**Figure 4:**
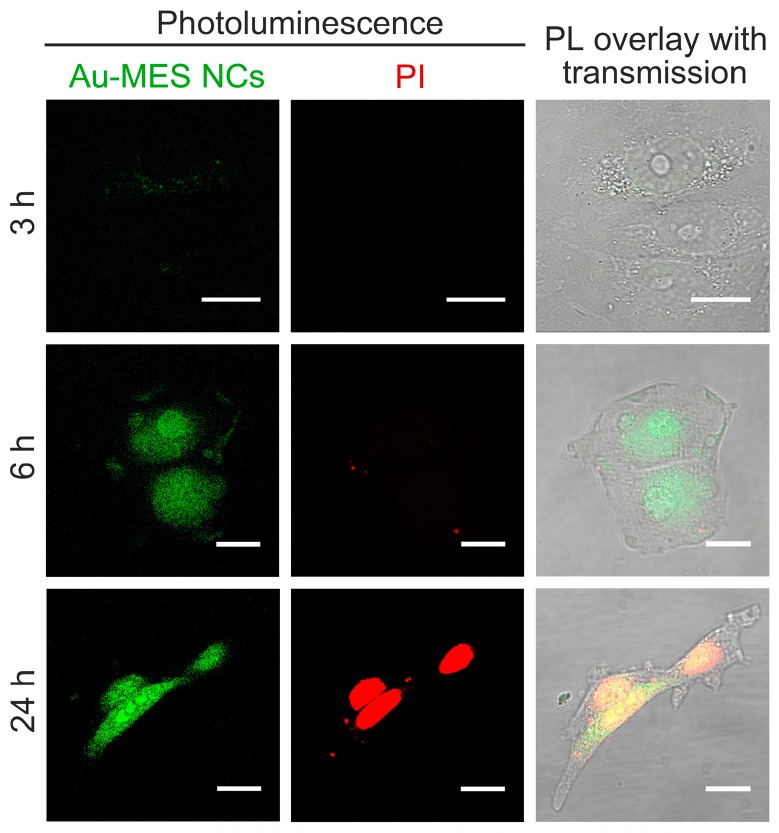
Accumulation of photoluminescent Au-MES NCs (λ_ex_ = 405 nm) in MCF-7 breast cancer cells after 3, 6, and 24 h of incubation (green photoluminescence). Red fluorescence represents propidium iodide (PI) stained non-viable cells (λ_ex_ = 488 nm). Yellow color in the merged pictures presents overlap of photoluminescence of Au-MES NCs and fluorescence of propidium iodide. Scale bar is 15 μm.

Accumulation of photoluminescent Au-MES NCs was very different from accumulation of BSA-Au NCs. After 3 h of incubation with Au-MES NCs solution, MCF-7 cells exhibited homogeneously distributed green photoluminescence (λ_ex_ = 405 nm) in 450–500 nm spectral region that was not observed in control group, only a few non-viable cells were stained with propidium iodide (PI) (Figure 4). After 6 h of incubation, the PL intensity inside the cells was higher. However, increased number of cells were stained with propidium iodide indicating increased cytotoxic effect. After 24 h of incubation the photoluminescence intensity increased even more, however, the propidium iodide staining revealed that almost all of the MCF-7 cells were non-viable. Simultaneous decrease of total number of the cells showed high cytotoxicity of Au-MES NCs solution. 

**Figure 5:**
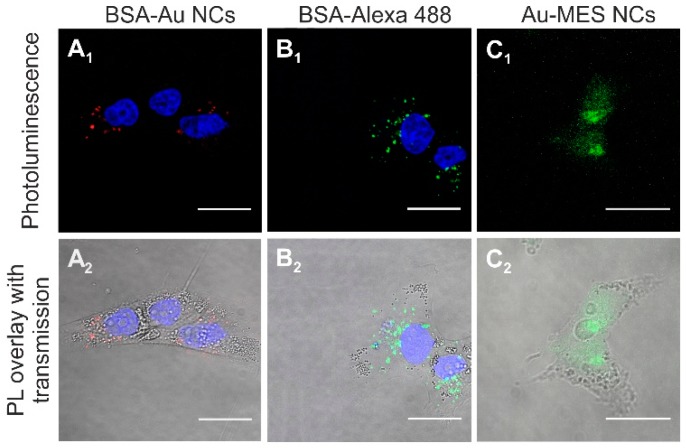
Accumulation of photoluminescent BSA-Au NCs (λ_ex_ = 488 nm) (**A_1_**,**A_2_**), BSA-Alexa 488 conjugate (λ_ex_ = 488 nm); (**B_1_**,**B_2_**), and photoluminescent Au-MES NCs; (**C_1_**,**C_2_**) in MDA-MB-231 cells. Cells were incubated with BSA-Au NCs and BSA-Alexa 488 conjugate for 24 h, with Au-MES NCs—for 6 h. In (**A_1_**,**A_2_**,**B_1_**,**B_2_**), nuclei were stained with Hoechst 33258 (λ_ex_ = 405 nm). Scale bar is 25 μm.

Accumulation of photoluminescent Au-MES NCs in MDA-MB-231 cells (Figure 5C_1_,C_2_) was very similar to the distribution in MCF-7 cells –the PL was homogeneous throughout the whole cell volume including cell nucleus, while both BSA-Alexa 488 conjugate and photoluminescent BSA-Au NCs were accumulated in vesicles at the perinuclear region (Figure 5A_1_,A_2_,B_1_,B_2_). 

**Figure 6:**
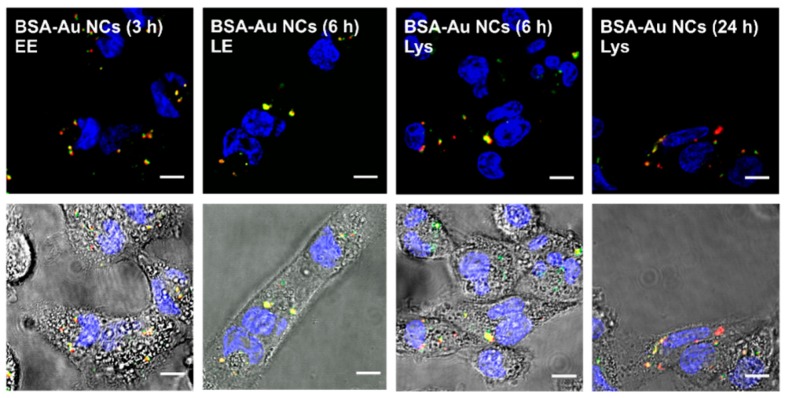
Intracellular distribution of photoluminescent BSA-Au NCs in early endosomes (EE), late endosomes (LE) and lysosomes (Lys). Yellow colour represents co-localisation of green fluorescent protein (GFP) labeled endosomal compartments and accumulated BSA-Au NCs. Scale bar is 10 μm.

As heterogeneous distribution of BSA-Au NCs in the cytoplasm of the cells was observed (Figure 2), BSA-Au NCs localization within endolysosomal pathway was investigated. MDA-MB-231 and MCF-7 cells were transfected with BacMam 2.0 system, and early endosomes, late endosomes and lysosomes were labelled with GFP. The spatial co-localization of BSA-Au NCs and endolysosomal compartments were evident from the appearance of yellow fluorescence combining green GFP and red BSA-Au NCs fluorescence. As it is shown in Figure 6, after 3 h of incubation BSA-Au NCs were observed in early endosomes that gradually matured into late endosomes and lysosomes at later points of time. Interestingly, as the incubation time increased BSA-Au NCs were found in all three endocytic compartments (data not shown) showing that endocytosis of BSA-Au NCs is a continuous process as long as there are NCs in the surrounding medium. Similar results were obtained in MCF-7 cancer cells (data not shown).

### 2.3. Cytotoxicity of Au NCs

**Figure 7:**
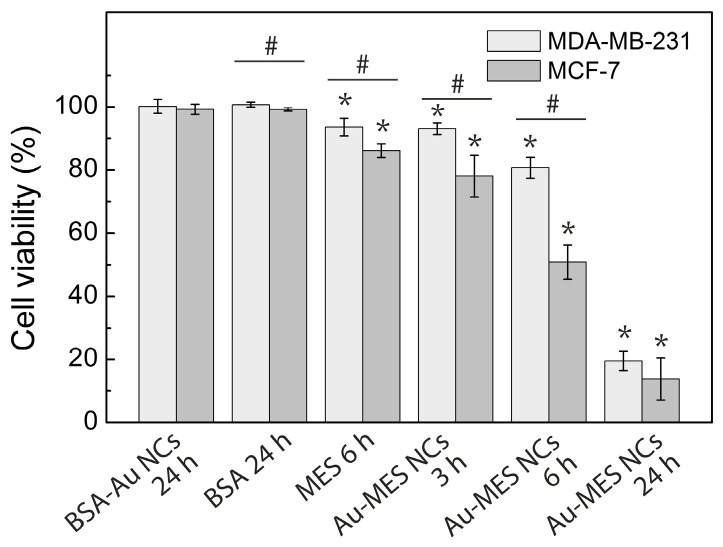
Cell viability of MCF-7 and MDA-MB-231 cells incubated with BSA-Au NCs, BSA, Au-MES NCs, and MES solutions. Error bars show the standard deviations. * indicates significant differences compared to the non-treated cells (Control) (*p* ≤ 0.05); ^#^ indicates significant differences between the MCF-7 and MDA-MB-231 cell lines (*p* ≤ 0.05).

To investigate the cytotoxicity of BSA-Au NCs and Au-MES NCs, cell viability upon exposure to these Au NCs was examined using advanced detection and accurate measurement automatic cell counting system ADAM-MC. As it is presented in Figure 6, cytotoxicity results showed no significant statistical difference of influence of BSA-Au NCs on viability of MCF-7 and MDA-MB-231 cells after 24 h of incubation. Incubation with BSA solution also did not affect cell viability. In contrast, 24 h incubation time was lethal in case of incubation with Au-MES NCs—cell viability of only 13.8% and 19.5% was calculated for MCF-7 and MDA-MB-231 cells, respectively (Figure 7). However, at shorter incubation times Au-MES NCs exhibited lower cytotoxicity: after 3 h of incubation with Au-MES NCs cell viability of MCF-7 and MDA-MB-231 cells was 78.1% and 93.1% respectively; after 6 h of incubation—50.9% and 80.7%. The cytotoxic effect of MES solution (1 M, pH 6.3) after 24 h of incubation was quite low, the cell viability of MCF-7 and MDA-MB-231 cells decreased to 86.1% and 93.6%, respectively. 

**Figure 8:**
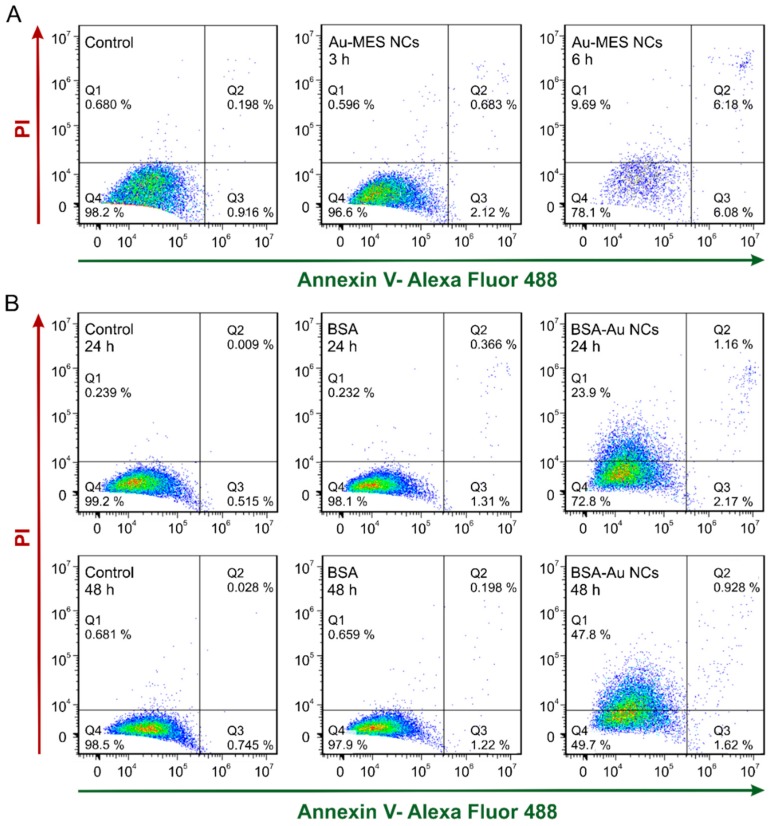
Apoptosis analysis of MDA-MB-231 cells after incubation with Au-MES NCs for 3 and 6 h (**A**); and BSA and BSA-Au NCs for 24 and 48 h (**B**) by flow cytometry, using Annexin V/ PI apoptosis assay. Only PI positive population (Q1) represents necrotic cells whereas only Annexin V positive population (Q3) are early apoptotic cells. Cells in late apoptosis take up both dyes (Q2). Q4 represent live cells. In the case of cells treated with BSA-Au NCs, population Q1 displays BSA-Au NCs positive cells.

To investigate whether Au NCs could cause cell apoptosis, we performed an Annexin V-Alexa Fluor 488/propidium iodide assay on MDA-MB-231 cells incubated with Au-MES NCs, BSA-Au NCs, and BSA. As expected, the highest extent of apoptosis was observed in cells treated with Au-MES NCs: after 3 and 6 h of incubation apoptosis rate was 2.80% and 12.26% respectively (Figure 8A). Incubation with Au-MES NCs for 6 h significantly decreased the number of viable cells (Q4, 78.1%), which is in agreement with the viability results (Figure 7). Moreover, 6 h exposure to Au-MES NCs also increased the amount of necrotic cells (Q1, 9.69%). Only minor induction of apoptosis was observed in cells upon treatment with BSA—as much as 1.68% apoptotic cells were detected (Figure 8B). BSA-Au NCs caused slightly higher apoptosis—3.33% and 2.55%—after 24 h and 48 h incubation, respectively.

### 2.4. ROS Generation of Au NCs in Cancer Cells

**Figure 9:**
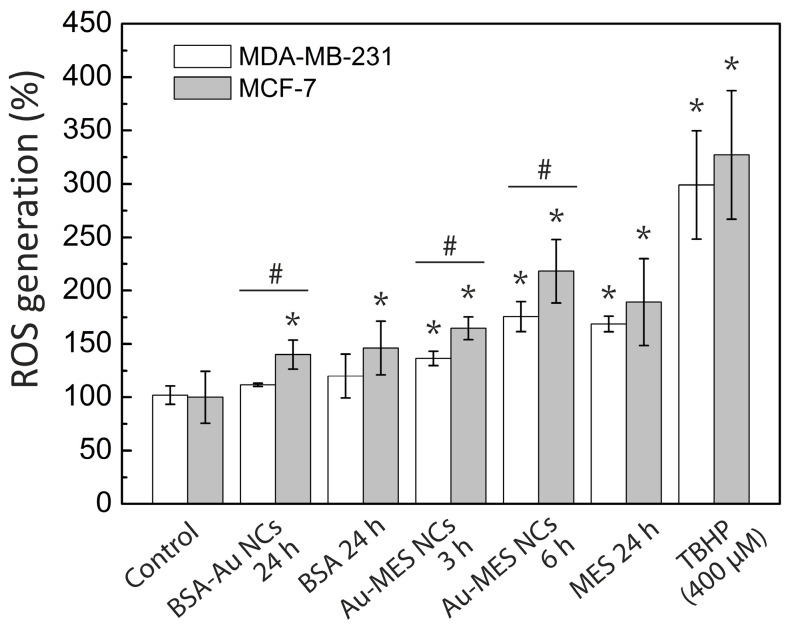
Reactive oxygen species (ROS) generation in MCF-7 and MDA-MB-231 cancer cells after treatment with BSA-Au NCs, Au-MES NCs, BSA, and MES. The data normalized according to the non-treated cells (Control), *tert*-Butyl hydroperoxide (TBHP) treated cells were taken as a positive control. Error bars show the standard deviations. * indicates significant differences compared to the non-treated cells (*p* ≤ 0.05); ^#^ indicates significant differences between the MCF-7 and MDA-MB-231 cell lines (*p* ≤ 0.05).

In order to determine whether generation of ROS caused by interaction of cells with Au-MES NCs plays a role in cell death induction, we used a flow cytometry assay to detect ROS in live cells. In this study, cells were treated with Au-MES NCs and BSA-Au NCs with following incubation with CellROX Green fluorescent ROS dye. Cells treated with 400 μg/mL of *tert*-Butyl hydroperoxide (TBHP) and CellROX Green were taken as positive control, and cells labelled only with fluorescent ROS dye represent non-treated control. The results showed that treatment with Au-MES NCs significantly increased intracellular ROS production (Figure 9). After 3 and 6 h of treatment, ROS generation in MDA-MB-231 cancer cells increased by 36.5% and 75.6% respectively in comparison with non-treated cells. The effect on MCF-7 cancer cells was even higher—after 3 and 6 h of treatment with Au-MES NCs, ROS generation increased by 64.7% and 118.2% respectively in comparison with non-treated cells (control). Incubation with MES solution (0.25 M) for 24 h induced increase in ROS production by 68.7% and 89.2% in MDA-MB-231 and MCF-7 cells, respectively. ROS production in the MDA-MB-231 cells treated with BSA-Au NCs for 24 h was not significantly different from the non-treated cells; however, in MCF-7 cells ROS generation increased by 40.1%. Similar results were obtained as the cells were incubated with BSA solution.

## 3. Discussion

Since photoluminescent gold nanoclusters hold tremendous potential to be employed in a wide range of biomedical applications [1,3,6], in the present study we analyzed and compared accumulation and toxicity of photoluminescent BSA-encapsulated and MES-capped gold nanoclusters in MCF-7 and MDA-MB-231 cancer cells along with their induced intracellular generation of reactive oxygen species. We have shown that photoluminescent BSA-Au NCs do not accumulate uniformly in both MCF-7 and MDA-MB-231 cells, only up to 73.5% of the MCF-7 cells and 68.8% of the MDA-MB-231 cells had internalized BSA-Au NCs after 24 h of incubation while BSA-Alexa 488 conjugate was observed accumulated in all MCF-7 and MDA-MB-231 cells after 24 h of incubation (Figure 3). Previously, we have also shown that the hydrodynamic size of BSA-Au NCs after the synthesis increased by approximately 2.5 nm (to 9.4 nm on average) compared to pure BSA [47] which could be due to the modification of the secondary structure of the BSA after labelling [48]. This transformation of the BSA structure could have caused difference of accumulation of BSA-Au NCs and BSA-Alexa 488 conjugate. However, both BSA-Au NCs and BSA-Alexa conjugate were observed accumulated in vesicles (Figure 5) indicating endocytotic uptake mechanism. Co-localization analysis confirmed endocytosis and demonstrated BSA-Au NCs localization in early endosomes after 3 h of incubation, followed by re-localization to late endosomes and lysosomes at later incubation times (Figure 6). Many authors have shown that BSA accumulates in cells via clathrin-mediated endocytosis and/or macropinocytosis [49,50,51]. The intracellular uptake of BSA-Au NCs reported in the literature is usually very low [52,53,54] and only conjugation with folic acid (FA) has shown to improve the uptake efficiency of BSA-Au-FA NCs in folic acid receptor (FR) positive cells through FR-mediated endocytosis [52,54]. However, the uptake mechanism of BSA-Au NCs has not been previously investigated.

In contrast, MES-capped photoluminescent gold NCs were distributed homogeneously inside MCF-7 and MDA-MB-231 cancer cells including nuclei (Figure 4 and Figure 5). It is known that some small, hydrophilic organic molecules—like sugars—can pass through the cell membrane by facilitated diffusion [55]. Transmembrane proteins create a water-filled pores that enable the molecule to pass through the cell membrane following its concentration gradient. The transport through the nucleopores of the cells is regulated by nuclear pore complexes which allow the diffusion of ions and small molecules (<40–60 kDa) across the nuclear envelope [56] and facilitate the receptor-mediated bidirectional transport of cargo molecules containing specific signals such as proteins, or RNAs [57,58]. There are several other reports showing that gold nanoparticles smaller than 1.4 nm in diameter can also pass through the cell membrane and even through nuclear membrane diffusely [59]. In the previous report, we have calculated that synthesized photoluminescent Au-MES NCs consist of ~9 gold atoms [45,60] showing that the size of the nanoclusters should be ~0.5 nm in diameter. Therefore, the small size of Au-MES NCs allows them to pass through the cell membrane and even through nuclear membrane diffusely. Large nanoparticles of the sizes up to 30 nm in diameter including BSA-Au NCs can also target nuclei, yet, in order to pass the nuclear envelope their surface has to be modified with nuclear localization signals [61,62]. Nanoparticles without proper nuclear localization signal are packed in endosomes upon entering the cells and cannot escape endocytic vesicles [61], therefore, BSA-Au NCs as well as BSA were observed accumulated in vesicles at the perinuclear region, but not inside the cell nucleus. Stable BSA template of Au NCs and inability to escape endosomes prevents the interaction of Au NCs with vital cellular components such as RNA and DNA resulting in no effect on cell viability. Because of the very same reason there was only minor increase in ROS generation in cases of cells treatment with pure BSA and BSA-Au NCs (Figure 9). Cytotoxicity assessments by other authors found BSA-Au NCs to be non-toxic to several other cell lines [6,53,54,63], yet there are papers showing decreased cell viability upon incubation with BSA-Au NCs [64]. Incubation with BSA alone has shown enhanced cell metabolism leading to higher cell proliferation [64], however, other authors report that high doses of BSA increase cell death rate [65]. Non-covalent bonding of BSA forming protein-coating has shown to reduce if not to completely overcome cytotoxic effect of NPs [64,66,67]. Only negligible increase in intracellular ROS generation induced by treatment with protein-encapsulated Au NCs have been reported by other authors [54] showing that proteins serve as a remarkable coating in terms of biocompatibility. On the contrary, presumably due to ultrasmall size leading to a more widespread intracellular distribution including cell nuclei, Au-MES NCs were found more biologically reactive than BSA-Au NCs. Such exposure increased the possibility of Au-MES NCs interaction with vital cell components, including cell DNA. Therefore, the toxicity (Figure 7) along with generation of ROS has increased greatly over time (Figure 9). Other components of the synthesis such as MES has shown low toxicity in high doses (Figure 7), HAuCl_4_ has also been reported to be not toxic in low doses [68]. The generation of ROS induced by nanomaterials can contribute to numerous biological stress responses and impair basic cellular functions leading to cell cycle arrest or even apoptosis [41,44]. Since ultrasmall (<2 nm) NCs exhibit extremely high surface area to volume ratio, it is no wonder that they induce increased intracellular ROS generation in comparison to their larger counterparts [69]. However, Tay et al. have shown that intracellular ROS generation is also ligand specific [37]. Heightened intracellular ROS levels are usually associated with catalytic activity of Au NCs. For instance, Pan et al. have shown oxidative stress caused by 1.4 nm triphenylphosphine monosulfonate coated Au NCs reduced upon co-incubation with thiol containing compounds showing direct contribution of Au NCs to increased intracellular ROS levels [69]. Oxidative stress is associated with lipid and protein oxidation leading to impairment of mitochondrial function and to further cell death. Annexin V/PI analysis confirmed the greater extent of apoptosis in cells treated with Au-MES NCs (12.3% apoptotic cells and 9.7% necrotic cells after 6 h) (Figure 8A) than in cells incubated with BSA-Au NCs or BSA alone (up to 3.3% and 1.7% apoptotic cells, respectively) (Figure 8B). Previous reports showed that both small molecule and protein stabilized Au NCs could cause apoptosis [33,64]. Pan et al. used Au NCs of 1.2 nm and 1.4 nm in size and detected that 6 h incubation with Au NCs leads to 20.6% apoptotic cells depending on NCs size. Yet this effect coincides with our results on Au-MES NCs capacity to induce apoptosis and necrosis. The BSA-Au NCs we studied are proven to be non-toxic, non-reactive and safe to use, on the contrary to Dong et al. [64]. However, it is difficult to evaluate the cytotoxicity of photoluminescent Au-MES NCs alone, as it is known that during the synthesis larger (up to 10 nm in size) non-luminescent, yet non-plasmonic nanoparticles are formed. 

The cytotoxic effect of Au-MES NCs was more significant on MCF-7 cells than on MDA-MB-231 cells, along with higher intracellular ROS generation, which is in agreement with the results reported by other authors showing that MDA-MB-231 cells are more resistant to treatment and exhibit properties of cancer stem-like cells [70,71].

## 4. Materials and Methods

### 4.1. Chemicals

Bovine serum albumin (V fraction), chloroauric acid (HAuCl_4_*H_2_O (99.9% purity)) and sodium hydroxide (NaOH, pellets (>99% purity) were purchased from Sigma-Aldrich (Taufkirchen, Germany) and used without further purification. MES (>98.0% purity) was purchased from Tokyo chemical industry (Zwijndrecht, Belgium). Deionized water was produced using ultrapure water system “MicroPure UV” (TKA, Lage/Lippe, Germany).

### 4.2. Synthesis of BSA-Au NCs

BSA-Au NCs were synthesized according to the previously reported procedure [18] with slight modifications [47]: briefly, aqueous HAuCl_4_ solution (5 mL, *c* = 5.27 × 10^−3^ M, 37 °C) was added to a BSA solution (5 mL, *c* = 7.53 × 10^−4^ M, 37 °C) under vigorous stirring. NaOH solution (0.5 mL, 1.0 M) was introduced 2 min later, and the reaction was allowed to proceed under vigorous stirring for 12 h at the temperature of 37 °C.

### 4.3. Synthesis of Au-MES NCs

The gold nanoclusters capped with MES were synthesized according to the previously reported synthesis protocol of Bao et al. [16] with slight modifications [45]: 5 mL of aqueous MES buffer solution (1 M, pH 6.3, the pH value was achieved using sodium hydroxide) was mixed with 1 mL of chloroauric acid solution (0.29 M). Synthesis was performed under vigorous stirring for 21.5 h at the temperature of 37 °C. After the synthesis solution of Au-MES NCs was centrifuged for 30 min at 6700× *g* with “MiniSpin plus” centrifuge (Eppendorf, Hamburg, Germany).

### 4.4. Spectrometric Measurements

Absorption spectra were measured with Varian Cary Win UV absorption spectrometer (Varian Inc., Mulgrave, Australia). Photoluminescence and photoluminescence excitation spectra were measured with Varian Cary Eclipse fluorescence spectrometer (Varian Inc., Mulgrave, Australia). For all spectrometric measurements quartz cuvettes of 1 cm optical path were used (Hellma Optik, Jena, Germany). The measurements were performed at room temperature.

### 4.5. Cell Culturing

The cell lines cultivated for in vitro experiment were human breast cancer cell lines MCF-7 and MDA-MB-231. MCF-7 cell line was purchased from the European Collection of Cell Cultures and MDA-MB-231 cell line was purchased from the American Type Culture Collection. Cells were cultured in cell growth medium (DMEM, Gibco, Waltham, MA, USA) supplemented with 10% (*v*/*v*) fetal bovine serum (FBS) (Gibco, Waltham, MA, USA), 100 U/mL penicillin, 100 mg/mL streptomycin, and 4 mM l-alanyl-glutamine (Biochrom, Berlin, Germany). Cells were maintained at 37 °C in a humidified atmosphere containing 5% of CO_2_. For the in vitro experiments, synthesized Au NCs solutions were filtered using a 0.02 mm syringe filter.

### 4.6. Transfection Assay

Transient transfection of MDA-MB-231 and MCF-7 cells was performed using Cell Light Reagent-GFP, BacMam 2.0 (Thermo Fisher Scientific, Waltham, Massachusetts, USA) according to the manufacturer’s recommendations. Briefly, the cells were seeded at a density of 1 × 10^5^ cells per well onto 12-well plate in complete growth medium. After cell attachment BacMam 2.0 reagent was added at a concentration of 60 particles per cell (PPC). Cell Light Early endosomes-GFP, BacMam 2.0 was used to label early endosomes (Rab5a-GFP expression), Cell Light Late endosomes-GFP, BacMam 2.0 was used to label late endosomes (Rab7a-GFP expression), and Cell Light Lysosomes-GFP, BacMam2.0 was used to label lysosomes (Lamp1-GFP expression). The cells were transfected for 72 h, then trypsinized and seeded into 8-chambered coverglass plates for fluorescence imaging experiments.

### 4.7. Treatment of Cancer Cells with Au NCs

For intracellular imaging studies cells were seeded into 8-chambered coverglass plates (Nunc Lab-Tek, Thermo Fisher Scientific, Campbell, CA, USA) with a density of 4 × 10^4^ cells/chamber and subsequently incubated at 37 °C in a humidified atmosphere containing 5% of CO_2_ for 24 h. The cells were treated with 56 mg/mL of BSA-Au NCs and 45 mg/mL of Au-MES NCs respectively then incubated under the same culture conditions for 24 h. To investigate accumulation of pure BSA, cells were incubated with 0.01 mg/mL of BSA-Alexa Fluor 488 conjugate (Invitrogen, Waltham, Massachusetts, USA) accordingly. After 24 h of incubation the cells were washed three times with Dulbecco’s phosphate buffered saline (DPBS, pH 7.0) (Sigma-Aldrich, St. Louis, MO, USA) and then incubated with 10 μg/mL of Hoechst 33258 (Sigma-Aldrich, Taufkirchen, Germany) solution for staining the nuclei of the cells before the subsequent examination with laser scanning confocal microscope. Non-viable cells were stained with propidium iodide (incubation with 1.5 μM PI solution for 10–15 min).

### 4.8. Imaging of Au NCs in Cancer Cells

The cellular uptake of Au NCs in cells was assessed using the Nikon Eclipse Te2000-U microscope (Nikon, Yokohama, Japan) with the confocal laser scanning system C1si (capable of 32-bit spectral imaging). Imaging was performed using a 60×/1.4 NA oil immersion objective (Plan Apo VC, Nikon, Yokohama, Japan). The BSA-Au NCs, BSA-Alexa, and propidium iodide were excited at 488 nm with argon-ion laser and Au-MES NCs and nucleus stain Hoechst 33258 were excited at 404 nm with diode laser. 

For investigation of uptake mechanisms of BSA-Au NCs, co-localization with endocytosis markers has been studied. GFP in transfected endosomes and lysosomes were excited at 488 nm with argon-ion laser and BSA-Au NCs were excited at 543 nm. Co-localization of BSA-Au NCs and GFP in superimposed images appear yellow.

The three-channel RGB detector (band-pass filters 450/17, 545/45 and 688/67 for blue, green, and red channels, respectively) was used. The cells were maintained at 37 °C in Microscope Stage Incubation System (OkoLab, Pozzuoli, Italy) in a humidified atmosphere containing 5% of CO_2_ (0.80 Nl/min O_2_ and 0.04 Nl/min CO_2_). Image processing was performed using the Nikon EZ-C1 Bronze version 3.80 and ImageJ 1.46 software (free non-commercial software developed at the National Institutes of Health, Bethesda, MD, USA).

### 4.9. Cell Viability Assay

Cell viability was evaluated with the ADAM-MC automated cell counter (Digital Bio Technology Co., Ltd, NanoEnTek Inc., Seoul, Korea). The cells were seeded on a 12-well plate at a density of 1 × 10^5^ cells/well and incubated for 24 h before the nanoparticles were applied. The old medium was replaced with fresh medium containing BSA-Au NCs (56 mg/mL) and Au-MES NCs (45 mg/mL), respectively. Cells incubated with medium alone were taken as control. After 24 h of incubation with BSA-Au NCs and 3, 6, and 24 h with Au-MES NCs, the medium with Au NCs was carefully aspirated and the cells were trypsinized and collected in the aspirated medium and then centrifuged at 200× *g* for 7 min and resuspended in 100 μL of phosphate buffered saline (PBS) (Gibco, Paisley, Scotland, UK) solution. 20 µL of cells suspension was mixed with 20 µL Accustain solution T and 20 µL Accustain solution N (Digital Bio, Seoul, Korea) for calculations of total and non-viable cells, respectively. The viability was automatically calculated by the ADAM-MC software after each measurement of the total cells and the non-viable cells.

### 4.10. Apoptosis Assay

MDA-MB-231 cells were seeded in 24-well plates at a density of 5 × 10^4^ cells per well in complete growth medium and let to adhere overnight. Cells were incubated with Au-MES NCs for 3 and 6 h, with BSA and BSA-Au NCs for 24 and 48 h. After the incubation cells were trypsinized, centrifuged at 200× *g* for 7 min, and washed with 1X Annexin-binding buffer (10 mM HEPES, 140 nM NaCl, 2.5 mM CaCl_2_) (Sigma-Aldrich, Taufkirchen, Germany) and centrifuged again. Cells were resuspended in 100 µL 1X Annexin-binding buffer, 4 µL Annexin V-Alexa Fluor 488 stock solution (Molecular Probes, Carlsbad, CA, USA) and 3 µL 100 µg/mL PI solution was added. Cells were incubated at room temperature for 15 min. After the incubation period, 300 µL of 1X Annexin-binding buffer was added and the samples were analyzed with flow cytometer. A minimum of 10,000 viable cells per sample were collected and analyzed. AnnexinV-Alexa Fluor 488 fluorescence emission was registered in FL1 (530/30), PI—in FL3 (670 LP). The data was analyzed with FlowJo (Tree Star, Inc., Ashland, Oregon, USA) or Accuri C6 software (Accuri Cytometers, Inc., Ann Arbor, Michigan, USA), electronic compensation between the red and green channels was performed to avoid spectral overlap. To exclude cell debris and doublets, cell population was chosen based on forward scattering (FSC) and side scattering (SSC). Dot plots were divided into quadrants according to Annexin V-Alexa Fluor 488 and PI-stained control. As BSA-Au NCs PL is registered in FL3, Q1 quadrant in BSA-Au-positive samples gives false-positive PI PL and was excluded from the analysis.

### 4.11. Quantitative Evaluation of Au NCs Accumulation in Cells and ROS Generation

The quantitative analysis of the cellular uptake of Au NCs and induced intercellular generation of ROS was assessed using flow cytometry assay.

For the evaluation of cellular uptake of BSA-Au NCs and BSA-Alexa, Fluor 488 conjugate cells were seeded into 24-well culture plates (BD Falcon, San Jose, California, USA) (5 × 10^4^ cells per well) and subsequently incubated at 37 °C in a humidified atmosphere containing 5% of CO_2_ for 24 h. The cells were then treated with BSA-Au NCs (56 mg/mL) and BSA-Alexa Fluor 488 conjugate (0.01 mg/mL) (Invitrogen, Carlsbad, CA, USA) for 3, 6, or 24 h. The cells were washed with PBS, trypsinized, and pelleted by centrifugation at 200× *g* for 7 min and resuspended in a final 100 μL volume of PBS solution for immediate analysis by flow cytometry.

For ROS generation analysis, after the treatment with Au NCs the cells were washed with PBS and incubated with 5 μM CellROX Green fluorescent ROS dye (Life technologies, Carlsbad, CA, USA) for 1 h and then washed with PBS, trypsinized, and prepared accordingly for the flow cytometry analysis. Upon oxidation, CellROX Green reagent binds to DNA and thus its signal is localized primarily in the nucleus and mitochondria. Cells treated with ROS inducer TBHP (Aldrich, Taufkirchen, Germany) for 1 h under standard culture conditions were used as a positive control (400 μM). Cells labelled only with fluorescent ROS dye represent non-treated cells (control). Flow cytometry was performed on Accuri C6 (Accuri Cytometers, Inc., Ann Arbor, MI, USA) flow cytometer. A minimum of 10,000 viable cells per sample were collected and analyzed. CellROX Green and BSA-Alexa were visualized using argon laser (488 nm) for excitation and 530/30 band pass filter for detection. Accumulation of BSA-Au NCs was evaluated using argon laser (488 nm) for excitation, and 670 nm long pass filter for detection. The data was analyzed with Flow Jo (Tree Star, Inc., Ashland, OR, USA) or Accuri C6 software (Accuri Cytometers, Inc., Ann Arbor, MI, USA).

### 4.12. Statistical Analysis

Data are shown as representative images or expressed as mean ± standard deviation (SD). Statistical significance of differences between studied groups (*n* = 3) was assessed using a two-tailed independent Student’s *t*-test at the 95% confidence level. Significance was represented as *p*-value < 0.05.

## 5. Conclusions

In summary, we have demonstrated that, despite some structural changes in protein conformation, BSA-Au NCs accumulate in cells in a similar manner as BSA alone, indicating an endocytotic uptake mechanism while ultrasmall Au-MES NCs were distributed homogeneously throughout the whole cell volume including cell nucleus. The cytotoxicity of BSA-Au NCs was negligible, demonstrating good biocompatibility of such BSA-protected Au NCs. However, incubation with high doses of either BSA or BSA-Au NCs increase intracellular ROS generation. In contrast, possibly due to ultrasmall size and thin coating layer, Au-MES NCs exhibited exposure time-dependent high cytotoxicity and higher reactivity, which led to highly increased generation of ROS and higher rate of apoptosis. Yet, further experiments are necessary to evaluate the input of other toxicity mechanisms such as genotoxicity, since ultrasmall size of Au-MES NCs imply possible interactions with DNA. The results demonstrate the importance of the coating layer to biocompatibility and toxicity of ultrasmall photoluminescent gold nanoclusters.
